# Negative symptoms in emerging psychosis: prognostic value and potential therapeutic targets

**DOI:** 10.1192/j.eurpsy.2026.10365

**Published:** 2026-07-31

**Authors:** S. Damiani

**Affiliations:** Department of Brain and Behavioral Sciences, University of Pavia, Pavia, Italy

## Abstract

**Abstract:**

Negative symptoms are a core dimension of schizophrenia spectrum disorders and are frequently detectable during emerging psychosis, including clinical high-risk states and first-episode psychosis. Accumulating longitudinal evidence indicates that early negative symptoms are among the strongest predictors of poor functional outcomes, limited symptomatic remission, and long-term disability, often exceeding the prognostic impact of positive symptoms. Despite their clinical relevance, negative symptoms remain difficult to treat and are frequently under-recognized in early intervention settings.

This talk aims to examine the prognostic value of negative symptoms in emerging psychosis and to discuss their relevance as potential therapeutic targets during the prodromal and early phases of illness. It will integrate evidence from prospective cohort studies, first-episode trials, and intervention research, with particular attention to the distinction between primary and secondary negative symptoms, measurement strategies, and clinically meaningful change.

The presentation will highlight that persistent negative symptoms in early psychosis are associated with poorer social and occupational functioning, reduced engagement with treatment, and less favorable recovery trajectories. While antipsychotics remain essential for the management of positive symptoms, their impact on negative symptoms is limited, underscoring the need for adjunctive and non-pharmacological approaches. Furhermore, evidence shows that early stages may represent a period in which negative symptoms are less entrenched and more responsive to interventions. The characteristics of this potential critical window and related therapeutic options will therefore be discussed.

**Disclosure of Interest:**

None Declared

